# A Latent Markov Model with Covariates to Study Unobserved Heterogeneity among Fertility Patterns of Couples Employing Natural Family Planning Methods

**DOI:** 10.3389/fpubh.2017.00186

**Published:** 2017-08-15

**Authors:** Fulvia Pennoni, Michele Barbato, Serena Del Zoppo

**Affiliations:** ^1^Department of Statistics and Quantitative Methods, University of Milano-Bicocca, Milano, Italy; ^2^Ambrosiano Centre for Natural Family Planning Methods (C.A.Me.N.), Milano, Italy

**Keywords:** expectation–maximization algorithm, latent stochastic process, natural family planning methods, predictive probabilities, state dependence

## Abstract

**Purpose:**

We use the historical data from the European Study of Daily Fecundability and we develop an algorithm to determine the fertile window in a woman’s cycle according to the rules of the C.A.Me.N. symptothermal method proposed by the Centro Ambrosiano Metodi Naturali. Our aim is to identify variables acting on the probability of conception by considering the fertile window and factors that cannot be explained by employing the observed covariates of individuals and couples.

**Methods:**

We adopt the latent Markov model with covariates tailored for data collected at times when a latent process detects the dependence across fertile periods of each woman’s cycle. We consider measurement errors, transitions between conception and non-conception, and the prediction of conception rate over the fertile windows.

**Conclusion:**

We find that the conception pattern is mainly related to sexual intercourse behavior during the fertile window and to previous pregnancies. For the cohort under study, we predict a steep decline in the average conception rate across fertile windows.

## 1 Introduction

We consider a dynamic latent variable model to assess the probability of conception within a fertile window of a woman’s menstrual cycle when a healthy couple decides to conform their sexual behavior to the rules provided by the natural family planning (NFP) methods.^1^ The model accounts for the longitudinal structure of the phenomena under study. In this context, it is useful to consider measurement errors which may arise because the woman does not correctly detect the quality of the cervical mucus secretions (CMS), or she does not correctly report the CMS type or the basal body temperature (BBT) on the chart which is used to record all the fertility signs.

The Latent Markov (LM) model with covariates that we employ in this work is well explained by Bartolucci et al. (1), and it may be considered a generalization of the latent class model, see, among others, Lazarsfeld and Henry (2), Lazarsfeld (3), and Pennoni (4). For a more comprehensive historical overview of the LM model see Bartolucci et al. (1) (Ch. 1) and Bartolucci et al. (5) (Sec. 2). Like the proposal of Dunson and Zhou (6), it accounts for the observed heterogeneity among women and unlikely to their work it also accounts for the time-varying unexplained heterogeneity between couples. It relies on the assumption that the response variables related to the conception history of the couple are conditional independent given a latent process which follows a Markov chain [Cappé et al. (7)] with transition probabilities between the two possible states of conception or non-conception.

The observed fertility pattern of the couple is associated with their observed covariates and with the latent process which explains variables influencing the variability among couples which we are unable to assess through data. This latent process that carries the dependence between fertile states at the different time points is assumed as a homogeneous Markov process of first-order meaning that it is dependent only on the immediately preceding state of the process and not on all the previous states. The conditional probability of the responses accounts for the observed covariates by a parameterization similar to that employed within the generalized linear model McCullagh and Nelder (8). In a similar way to our proposal, the LM model has been considered to relate the pregnancy probability to employment by Bartolucci and Farcomeni (9).

Several Bayesian statistical models have been proposed to identify the days in the menstrual cycle with the highest probability of fertility according with time of the sexual intercourse as well as to predict the conception probability, see, among others, Bigelow et al. (10), Scarpa et al. (11), and Scarpa and Dunson (12–14). They model the BBT by allowing for the hierarchical structure of women and cycles. Other studies tend to identify groups of individuals with similar patterns of change in the CMS such as the proposal of Bassi and Scarpa (15) where they successfully apply the latent class model to find homogenous groups of women see, among others, Biemer (16).

A peculiarity of our proposal is the identification of the fertile window which is not a putative window of 12 days, as frequently used in the literature of the NFP methods for the estimates of daily fecundability but it is detected for each woman in each cycle through the rules of the NFP method known as the C.A.Me.N. symptothermal method. With the proposed LM model, we account for the dependence between cycles and we detect the unexplained heterogeneity among women while considering the sexual intercourse behavior of the couple as a covariate in the model. This is a novelty with respect to the proposal made by Colombo and Masarotto (17) and with respect to the proposal of Zhou et al. (18), which accommodates only for random effects in the model. We avoid the addition of model parameters related to BBT and to CMS and we account jointly for the unobserved confounders among women and for baseline characteristics such as couple-specific features and previous births.

In this context, the use of a joint model such as that proposed by McLain et al. (19) may be useful when the informative censoring is available and allows one to gain some knowledge of the survival probabilities. However, our proposal does not require the strong parametric assumptions employed by McLain et al. (19) and we can predict the probability of conception over the fertile windows by taking into account the available covariates.

The remainder of the paper is organized as follows. In Sec. 2, we provide details of the data collected in the European Study of Daily Fecundability (ESDF) which we use as illustrative example. In Sec. 3, we introduce the statistical model to investigate how the probability of conception is dependent across cycles, we outline some model features such as the estimation method and we summarize the results of the model estimation to the available data. In Sec. 4, we discuss the results and we suggest some developments for further research.

## 2 Prospective Pregnancy Study

The NFP methods are used to identify the few days in a woman’s menstrual cycle when conception can occur. They are based on knowledge of the biological and physiological processes of the female menstrual cycle. The couples are defined as “spacers,” if they intend to postpone pregnancy, or “limiters,” if they intend to avoid pregnancy. For a research on the effectiveness of the fertility awareness based methods related to the probability of avoiding pregnancy, see, among others, Frank-Herrmann et al. (20) and references therein.

From among the NFP methods we consider the C.A.Me.N. (Centro Ambrosiano Metodi Naturali) *symptothermal method* first suggested by Barbato and Bertolotti (21). This method aims to identify the days in a woman’s menstrual cycle when conception is potentially feasible. The name “symptothermal” comes from “symptoms” and “thermal” or “thermometer” since it is based on the perceived and observed fertility indicators that reflects the normal sequence of hormonal changes characterizing the cycle, see, among others, Stanford et al. (22). The perceived CMS that predict the ovulation, the observed changes of the BBT and the felt changes of the cervix are the main fertility indicators considered by the NFP methods.

The female fertile period is not so straightforward to seek for as the ovulation may suddenly come early or be postponed due to psychological changes (as stress, anxiety, sadness, happiness, or anger) or to endocrine disorders (dysthyroidism, hyperprolactinemia, etc.). Moreover, the biological fertile window is determined by the survival of the sperm in the female genital tract and by the survival of the ovum. The average lifetime of oocyte after ovulation is less than 24 h. Studies on intracytoplasmic sperm injection suggest that mature human ova have a more limited optimum fertilization window than previously appreciated (hours instead of days, see Yuzpe et al. (23)). Nevertheless, a period of several days is considered to be fertile due to the fact that it is also necessary to consider the average lifetime of sperm cells, which survive for some days in a fertile mucus (even for up to 6 days in certain studies, see, among others, Katz (24)).

For these reasons, the debate on the effectiveness of the NFP methods is still open and the literature on this field is growing. Ogino and Knaus (25) working separately in Japan and in Austria, respectively, were the first to place the ovulatory period between the 12th and 16th day before menses. Nowadays, numerous NFP methods exist differing as to the rules and the markers to find the fertile window. We mention the Billings ovulation method [Brown et al. (26)], the Creighton model method,^2^ and the TwoDay algorithm Sinai et al. (27), which consider only the observed CMS in order to determine the fertile window, Keefe method (28), only accounts for the modifications of the neck of the uterus through the cycle.

The requirements of C.A.Me.N. symptothermal method to determine the fertility time are explained in specialized centers where fertility awareness providers help women to classify the CMS. The intensity of the secretions rises according to estrogen when the ovulation is approaching and it is scored on a 5-point ordinal scale ranging from 0 to 4 on the basis of perception and appearance, as reported in Table 1. During the fertile days, CMS is transparent, stretchy and watery and the cervix is completely open and particularly soft in comparison to the infertile days. The BBT shift is a marker of the end of the fertile days within the cycle. Its shift confirms the detection of the ovulation phase. The latter is established through the three over six rule [Burrett and Marshall (29)], i.e., the first time in the cycle that three temperatures are subsequently recorded with higher levels than the immediately previous six temperatures, see, for more details, Marshall (30).

**Table 1:** Classification of cervical mucus symptoms and secretions.

Levels	Feeling	Appearance	Secretion
0	Not registered	Not registered	Not registered
1	Dry or nothing felt	Nothing seen	None
2	Damp	Nothing seen	None
3	Damp	Yellowish and sticky	Secretions
4	Wet and slippery	Transparent, stretchy, and watery	Secretions

The menstrual phases according to the C.A.Me.N. symptothermal method are summarized in Table 2. To apply the above methods the women must be able to perceive changes in the vulva (“feel, then seen”), before observing the CMSs and they must learn to evaluate adequately the changes in the neck of uterus. Therefore, relying on the observational skills of her body a woman can learn to detect the fertile window within each cycle irrespective of the menstrual cycle length.

**Table 2:** Menstrual phases according to the rule of the C.A.Me.N. symptothermal method.

Phase	Description
Pre-ovulatory infertile phase	From the first day of menses (if the previous cycle was ovulatory) until the last dry day (no mucus secretions)
Fertile phase	From the first day of felt or detected mucus till the evening of the 3rd day of high temperatures after the mucus peak
Post-ovulatory sterile phase	From the evening of the third day of high temperatures after the mucus peak till the last day of the cycle

The couple is also trained to make a daily record of the biological fertility indicators on a suitable chart in order to learn how to detect the initial and end phases of the fertile period. The training is given to the couple so that both male and female can be aware of their fertility and share a common responsibility. In this way, they may decide to achieve pregnancy by practicing sexual intercourse during the fertile period or to avoid or postpone pregnancy by abstaining from sexual intercourse on the detected fertile days. According to a recent international survey conducted among couples [Unseld et al. (31)], they are mostly satisfied with the NFP method since it helps to acquire deeper self-knowledge and also to strengthen their relationship.

### 2.1 Data Description

In the following, we use the historical data from the European Study of Daily Fecundability (ESDF) [Colombo and Masarotto; Dunson et al. (17, 32)] which have been collected in a prospective way by a team coordinated by Prof. Colombo^3^ while working at the Department of Statistical Science at the University of Padua.

As stated by Colombo and Masarotto (17) “*The research protocol was reviewed and approved by the Institutional Review Boards of Fondazione Lanza (Padua, Italy) and Georgetown University (Washington, DC, USA).”* The study design is hierarchical and multilevel and it has been a pioneer study in this field due to the rigorous protocol which was applied. For example, cycles in which a single act of protected sexual intercourse was registered, were excluded from the study, so that the registered patterns do not include protected and unprotected sexual intercourse.

The ESDF data were collected from 1992 to 1996 in seven European centers located in Milan, Verona, Lugano, Düsseldorf, Paris, London, and Brussels with the aim of determining fecundability estimates among healthy couples. A total of 782 couples meeting the following inclusion/exclusion criteria were enrolled: married or in a stable relationship and instructed to the NFP methods. The enrolled women had to be aged between 18 and 40, had to have at least a menstrual flow after cessation of breastfeeding or after delivery, and had not to be taking drugs affecting fertility on entrance to the study. It was required that there were no infertility problems in the couples. Some initial information on the couples was collected, such as dates of births, date of marriage for married couples, number of previous pregnancies, date of the last delivery or miscarriage, date of the end of breastfeeding, and date when the last oral contraceptive was taken. Furthermore, the sex of the babies born during the study is supplied.

Before entering the study, the couples were trained on how to record the various phases and the instances of the sexual intercourses. The women, in each menstrual cycle, recorded daily the BBT, taken on awakening in the morning before engaging in any activity. They also recorded the changes in CMS, in accordance with daily mucus symptom described in Table 1 as well as any disturbances such as illness. We deleted the cycles for which there was not information to determine the fertile period according to C.A.Me.N. symptothermal method as described in Sec. 2, e.g., cycles without BBT values and/or CMS levels. Therefore, starting from the original 7,288 menstrual cycles and 782 women, we ended up with a total of 5,868 cycles and 758 women. By starting from the daily information collected from the chart, we implemented a suitable algorithm to determine the fertile window from the data in accordance with the C.A.Me.N. rule. The algorithm also accounts for women reporting values which are not strictly in accordance with the rule. By excluding the cycles with no reported acts of intercourse within the fertile period we ended up with 2,786 cycles and 673 women.

As reported in Table 3, at the first entry in the study, women were on average 30 years old, while men were on average 32 years old; 52% of the women did not have previous pregnancies and 30% of them used hormonal contraception. During the study, there were 408 pregnancies and the overall percentage of pregnancy is 58%. Table 4 shows the drop-out reasons according the number of the entries in the study. Most of the couple’s entry in the study was only once, 55 twice. Pregnancy and miscarriage are the main reasons to drop-out of the study. It is to be kept in mind that a woman can again enter into the study after her infertile phase due to pregnancy, precisely at the second cycle after the pregnancy. There were 374 pregnancies at the first entrance and 31 pregnancies at the second entrance. There were 39 women who had a miscarriage before 60 days from the first entry, and only 4 women had a miscarriage at the second entry. According to Table 5, for 13% of the women, we can observe more than 7 menstrual cycles. As shown in Table 6, the majority of women were resident in Milan or in Verona. The Verona and Lugano centers have the highest percentage of pregnancy. The lowest percentage of pregnancy is indeed observed in Düsseldorf.

**Table 3:** Descriptive statistics of the data.

	# women	% women
**Event before entry in the study**		
Miscarriage	62	9.21
End of breastfeeding	206	30.61
Birth of a child	55	8.17
None	350	52.01
**Previous pregnancies**		
0	350	52.01
1	144	21.40
2	88	13.08
3	52	7.73
4	28	4.16
5	6	0.89
6	3	0.45
7	1	0.15
8	1	0.15
**Hormonal contraception**		
Yes	199	29.57
No	472	70.13
Missing	2	0.30
**Number of pregnancies**		
0	283	42.05
1	373	55.42
2	16	2.38
3	1	0.15
**Pregnancies**		
Cycle 1	111	16.49
Cycle 2	77	11.44
Cycle 3	63	9.36
Cycle 4	57	8.47
Cycle 5	25	3.71
Cycle 6	18	2.67
Cycle 7	17	2.53
Cycle 8	7	1.04

	**Mean**	**SD**

*Woman’s age (years)*	30	3.98
*Man’s age (years)*	32	4.72
*Average length of the fertile period (days)*	12	3.12
*Average number of intercourses*	3	1.99

**Table 4:** Number of women according to drop-out reasons and number of entries in the study.

Drop-out	# entries in the study				Total
	1	2	3	4	
Pregnancy	374	31	3	0	408
Miscarriage	39	4	0	0	43
End of the study	52	6	1	1	60
Other reasons	208	14	1	0	223
Total	673	55	5	1	–

**Table 5:** Number and percentage of women by menstrual cycles.

# cycles	# women	% women
1	174	25.85
2	120	17.83
3	100	14.86
4	81	12.04
5	44	6.54
6	31	4.61
7	34	5.05
8 or more	89	13.22

**Table 6:** Number and percentage of women and percentages of pregnancy for the women enrolled in each of the NFP centers.

	# women	% women	% pregnancies
Verona	197	29.27	72.59
Milan	228	33.88	50.88
Lugano	13	1.93	92.31
Paris	92	13.67	56.52
Düsseldorf	82	12.18	42.68
London	37	5.50	51.35
Brussels	24	3.57	54.17

## 3 Model Formulation and Estimation Procedure

We consider the binary event of conception (“yes” or “no”) and we denote as *Y*^(^*^t^*^)^, *t* = 1,…, *T* the random variable equal to 1 for the event of conception during the fertile window *t* or to 0 otherwise. The fertile window is determined according to the rules of the C.A.Me.N. *symptothermal method* as explained in Sec. 2. We collect these random variables into ***Y*** = (*Y*^(^*^t^*^)^,…, *Y*^(^*^T^*^)^) denoting the response vector for each woman. We use the lowercase to denote realizations of random variables and vectors so that, for instance, ***y*** denotes a realization of ***Y***. Let also ***X***^(^*^t^*^)^ be the corresponding column vector of the time-fixed and/or time-varying covariates at time *t*. Note that if available, couple’s specific categorical, ordered, or continuous covariates can be included into the model.

We account for the time-varying unexplained heterogeneity as meaning that any response variable *Y*^(^*^t^*^)^ depends also on an occasion-specific latent variable *U*^(^*^t^*^)^. We assume that *Y*^(^*^t^*^)^,…, *Y*^(^*^T^*^)^ are conditional independent given ***U*** = (*U*^(1)^,…, *U*^(^*^T^*^)^) which is a latent process assumed to follow a first-order Markov chain with two state space since we conceive the latent states as the true states of the chain and the observed values as “proxy” states. It is to be kept in mind we are interested in modeling the unexplained heterogeneity, i.e., that the observed pregnancy probability cannot be explained only on the basis of the observed covariates. The observed responses related to pregnancy are conditionally independent across the fertile periods given this latent process and the observed covariates.

The model parameters are related to the measurement model, which concerns the conditional distribution of the response variables given the latent process, and to the latent model, which concerns the distribution of the latent process. The parameters of the measurement model are the following conditional response probabilities.

$$\phi_{y|ux}^{\left(t\right)}=p(Y^{\left(t\right)}=y|U^{\left(t\right)}=u,X^{\left(t\right)}=x),\;y=0,1,$$

where *Y*^(^*^t^*^)^ is the single response variable at time occasion *t* and ***X***^(^*^t^*^)^ is the vector of covariates associated with the response at the same time occasion.

The parameters of the latent process are the initial probabilities of each state and the transition probabilities between states which are denoted as

(1) $$\pi_{u}=p(U^{\left(1\right)}=u),\;u=1,2$$

(2) $$\pi_{u|\bar{u}}=p(U^{\left(t\right)}=u|U^{\left(t-1\right)}=\bar{u}),\;t=2,\ldots ,T,\;\bar{u},\;u=1,2,$$

where *U*^(1)^ denotes the latent process at the first time occasion. The parameters of the latent process may be affected by the covariates but in the context of the applicative example the covariates are acting on the observed response variables as we are mainly interested in characterizing the latent process according to the unexplained heterogeneity between couples. We use a parametrization which makes the latent states interpretable in terms of a possible conception. The conditional response probabilities are parametrized according to the following generalized logit that is an extension of the random parameter logit model where we assume a Bernoulli distribution for the response variable with a certain “success” probability of the following type:

(3) $$\phi_{y|ux}^{\left(t\right)}=\text{log}\frac{p(Y^{\left(t\right)}=\text{1}|U^{\left(t\right)}=u,X^{\left(t\right)}=x)}{p(Y^{\left(t\right)}=\text{0}|U^{\left(t\right)}=u,X^{\left(t\right)}=x)}=\mu_{y}+\alpha_{u}+x_{\mathrm{it}}^{\prime }\;\beta ,\;$$

for each couple, *i* = 1,…, *n* and *t* = 1,…, *T*, where *μ_y_* is the coefficient of the cut-point related to the response variable when equal to 1, *αu* represents the support point of the latent process when it is equal to the first latent state and it helps to define how this probability varies according to the two states of the chain, and ***β*** is the vector of the regression coefficients for the observed covariates in ***x***. The main interest is in the parameters ***β*** which allows us to measure the influence of each covariate on the conception probability.

Let ***θ*** denote the vector of all the model parameters that are estimated by maximizing the model log-likelihood. The latter is considered given a sample of *n* independent couples that provide the response vector ***y***_1_,…, ***y****_n_* and a corresponding vector of covariates ***x***_1_,…, ***x****_n_* as

$$\ell (\theta )=\sum_{i=1}^{n}\;f(y_{i}|x_{i})$$

where *f* (***y***|***x***) denotes the mass probability for the manifest distribution of the responses given the observed covariates.

The maximum likelihood estimation of the model parameters is carried out by employing the Expectation–Maximization algorithm [Dempster et al. (33)]. In this context, we have to consider the *complete data log-likelihood*. The complete data are represented by observed responses and by the sequence of latent states for each unit at each time occasion. Therefore, they could be computed only if we knew the joint frequencies of the covariate configuration, of the response configuration and of the latent process. The EM algorithm is employed to recover iteratively the above data and simultaneously maximize the complete data log-likelihood which is given by

(4) $$\ell^{*}(\theta )=\sum_{i=1}^{n}\{\sum_{t=1}^{T}\;\sum_{u=1}^{2}\;\sum_{y=0}^{1}\;a_{\mathrm{iuxy}}^{\left(t\right)}\text{ log}\phi_{y|ux}^{\left(t\right)}+\sum_{u=1}^{2}\;b_{\mathrm{iu}}^{\left(1\right)}\pi_{u},+\sum_{t=2}^{T}\;\sum_{u=1}^{2}\;\sum_{\bar{u}=1}^{2}\;b_{iu\bar{u}}^{\left(t\right)}\pi_{u|\bar{u}}\},$$

where $b_{iu}^{\left(1\right)}$ is a dummy variable for unit *i* in component *u* at the first occasion, with reference to the same occasion and the same unit, $b_{iu\bar{u}}^{\left(t\right)}$ is a dummy variable equal to 1 if this unit moves from state $\bar{u}$ to state *u* at occasion *t*, whereas $a_{iuxy}^{\left(t\right)}$ is equal to 1 if the unit is in state *u* and provide response *y* and covariate configuration ***x*** at occasion *t*. Since the frequencies in equation 4 are not known they are iteratively imputed. The E-step and the M-step of the EM algorithm are alternated at each iteration until convergence in the incomplete data log-likelihood ℓ(θ):

– E-step: compute the expected value of the frequencies *a_u**x**y_*, *b_u_*, and $b_{u\bar{u}}^{\left(t\right)}$ given observed data and the current parameter estimates of the complete data log-likelihood. Some recursions [Baum et al.; Welch (34, 35)] are needed to compute the manifest distribution (measurement model) of the response variables given the observed covariates and the latent variables;
– M-step: update the parameter estimates by maximizing the expected value of $\ell^{*}(\theta )$ computed on the basis of the expected frequencies obtained at the end of the E-step.

A detailed description of the steps above is available in Bartolucci et al. (1) (see the following parts of the book: Appendix 1 of Ch. 3, and Sec. 5.6 of Ch.5). The initialization of the algorithm is made by applying a deterministic rule or by using a random initialization modifying the deterministic starting values. This is an important feature since as in other mixture models (see, among others, McLachlan and Peel (36)), a proper estimation procedure should try to avoid local solutions and it should explore the entire parameter space since the likelihood may be multimodal. Therefore, within our approach both types of initial values are considered and we compare the values of the log-likelihood to choose the highest one among the observed values as the final estimate of the model parameters. For a more detailed description of the initialization method for the algorithm see also Bartolucci et al. (5) (Sec. 6.1.1).

Then, we take the parameter vector that at convergence with the highest value of ℓ(θ) denoted by $\ell (\hat{\theta })$. This approach is also important to get reliable standard errors which are determined by the estimated information matrix. Generally its full rank denotes that the model is locally identified and this may be cumbersome to verify. The standard errors are obtained as the square root of the inverse of the estimated Fisher information matrix calculated on $\hat{\theta }$.

In the context at hand the number of states is *a priori* defined. Prediction of the entire sequence of the latent states for a certain couple on the basis of their observed conception pattern can be obtained by maximizing the estimated conditional posterior probabilities of the latent variable given the covariates and the observed response pattern for each couple. In this way, a prediction of the changes between latent states for each couple at each time occasion can be achieved. These posterior probabilities are computed at the last iteration of the EM algorithm for each time occasion and each state according to every observed configuration of conception on the basis of the observed covariates. Additional technical details for the computational issues are provided by Bartolucci et al. (1) (see Sec. 7.5 of Ch. 7). See also Pennoni and Romeo (37) for a comparison between the prediction obtained within the LM model with covariates and the latent growth curve model which is another model for the analysis of longitudinal data proposed by Muthén (38).

### 3.1 Results

We applied the model presented above to the data described in Sec 2. The response variable is equal to 1 to indicate the event of conception within each fertile period, *t* = 1,…, *T*, and to 0 otherwise. We used all the available covariates collected in the prospective study: *woman’s age* and *man’s age* which are collected upon entrance to the study, *average length of the fertile period, average frequency of sexual intercourse in the fertile period, previous pregnancies* (equal to 1 for previous pregnancy or more), and *hormonal contraception* before entering the study (equal to 1 for using contraception). As shown in Sec. 2, all the available covariates may represent a source of variability of the response variable and we argue that there is also heterogeneity due to unexplained factors affecting fertility which may be due for example to the lifestyle.

By fitting the proposed model with the R Core Team (39) LMest package [Bartolucci et al. (40)] for an increasing number of fertile windows we noticed that the model with the first four fertile windows resulted in the highest log-likelihood value at the maximum. In the following, we report the results obtained with this number of fertile windows for which the estimated model shows a log-likelihood equal to −933. The estimated model parameters are those related to the parameterization adopted for the conditional probabilities of the responses as in equation 3 and those characterizing the latent model as in equations 1 and 2. The parameters for the measurement model are: the cut-point ${\hat{\mu }}_{y}$ equal to −0.079, the estimated support point, indicating that the second state is identified as the pregnancy state, and the coefficients related to the observed covariates collected in ***β*** which are displayed in Table 7. The sign of the coefficients shows that the probability of conception is negatively related to male and female age. However, it can be noticed that the magnitude is low and men’s age is not significant neither at 10%. The difference in age between males and females when added into the model is not significant neither at 10%. It may be due to the fact that the model is conceived to give importance to the biological age and not the actual age in years. The average length of the fertile period is slightly negatively associated with fecundity and it is significantly different from zero. The average frequency of sexual intercourse is positively associated with the probability of clinical conception as well as the number of previous pregnancies which have both a positive sign and are significantly different from zero. The absence of hormonal contraception used before entry in the study is positively associated with fecundity but the coefficient is not significant neither at 10%. Therefore, our principal conclusion is that the average frequency of sexual intercourse within the fertile window is associated with higher fecundability and parous women are more fecund than nulliparous women.

**Table 7:** Estimates of the regression coefficients affecting the conditional probability of clinical pregnancy given the latent process under the LM model with two latent states (*significant at 1%, ***significant at 10%).

Covariate	Estimate	Standard error
Woman’s age	−0.0415***	0.0229
Man’s age	−0.0212	0.0195
Average length of the fertile period	−0.0653*	0.0210
Average frequency of intercourse	0.2058*	0.0377
Number of previous pregnancies	0.3869*	0.1333
Absence of hormonal contraception	0.0299	0.1340

The estimated parameters characterizing the latent structure of the model are the probability of belonging to each state at the first fertile period and the probabilities to change or retain the state at the following fertile periods. At the first fertile window, the estimated probability of fecundity (${\hat{\pi }}_{2}$) is equal to 0.373, meaning that we expect at least 37% of women to become pregnant at the first observed fertile window. This is higher than the fraction observed in the sample since the estimation is referred to the whole population when we account for measurement errors and unobserved confounders giving rise to the observed data. The estimated transition probabilities (${\hat{\pi }}_{u|\bar{u}}$) are reported in Table 8. After the first fertile window, the probability to retain the same state conditional on the previous state is higher for those couples in state 1 indicating infecundity (0.9 versus 0.8). The probability of conception (state 2) given that the couple has been previously infecund (state 1) is equal to 0.122.

**Table 8:** Estimates of the transition probabilities between non-conception (1) and conception (2) states referred to the LM chain of the estimated model.

	${\hat{\pi }}_{u|\bar{u}}$	
$\bar{u}$	*u* = 1	*u* = 2
1	0.8771	0.1229
2	0.2066	0.7934

**Figure 1:**
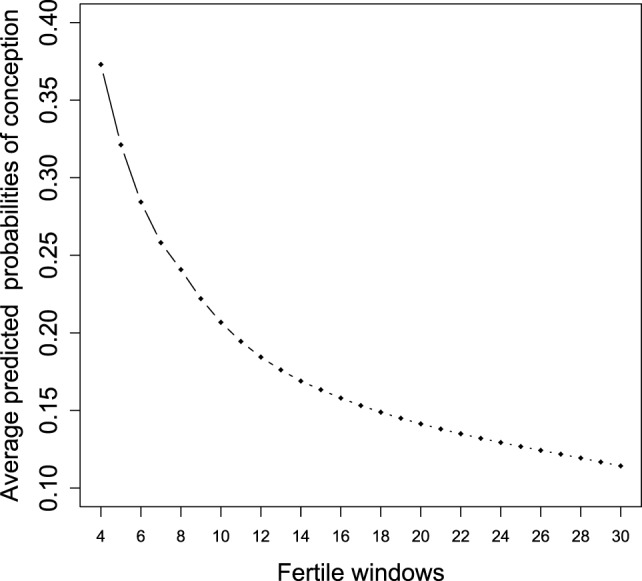
Average predicted probabilities of conception (state 2) in accordance with the LM model estimated for an increasing number of fertile windows.

Figure 1 shows the changes in the average predicted probabilities as described above for the latent state 2 (conception) when an increasing number of fertile windows is added into the LM model. In this way, we dispose of the predicted decline rate in fecundity for healthy couples across fertile windows by accounting for all the available covariates. These estimates have been gained as the log-likelihood of the proposed LM model is made by product of independent Bernoulli outcomes. Therefore, it is possible to estimate each model by adding an observed fertile window and obtaining comparable estimated average joint posterior conditional probabilities for each state of interest. The predicted pattern of decline is in accordance with that obtained by Zhou et al. (18) related to a different cohort where the expected probabilities were slightly lower than our estimates. Their prediction was made according to a random effect model only up to five cycles from a study conducted in North Carolina where the urine specimens were used to establish ovulation.

## 4 Discussion

We consider that there can be many factors influencing fecundity which are not observed and that women may commit errors in reporting the BBT values and the CMS levels when using NFP methods. We model the conditional distribution of the binary response variables indicating conception or non-conception to a latent Markov process of first order and to the observed covariates by means of a generalized logit model accounting for the woman and couple specific time-varying and time-fixed covariates. The model allows us to estimate the probability to move between the fecund and infecund state or to retain the same state. We estimate the average posterior predicted probabilities of fertility according to the available covariates in order to dispose of the predicted fertility rate of decline over time.

We show that the probability of conception within each fertile period is positively associated with the average number of previous pregnancies, with the average frequency of sexual intercourse as well as with the average length of the fertile period. The age of the woman slightly influences in a negative sense the probability of conception, and man’s age is not significant, the difference in age between them does not influence this probability. This may be because we are considering the fertile windows to measure time in the model and not the woman’s cycles. Hormonal contraception used before entry in the study does not affect the probability of becoming pregnant.

Nowadays, in Western societies, the reproductive age has shifted toward higher levels than 40 years. Therefore, more data need to be collected on healthy couples practicing the NFP methods so that it could be possible to investigate if there are different fertility patterns for less young couples and if this variability exists among towns or countries. Another aspect which, with more available data, it will be feasible to investigate with the proposed latent Markov model is the comparison of the efficacy of slightly different ways to open the fertile window within the C.A.Me.N. symptothermal method. In fact, a less restrictive rule of the method than that applied here states that the fertile window may also be open when the CMS is yellowish and sticky (level three instead of level two, see Table 1).

Other fecundability patterns can be investigated by considering the time-varying values of the covariates for each fertile window. In the latter case, the predicted probabilities could be compared according to parous and nulliparous women’s ages. Moreover, the estimated predicted trajectories of these probabilities can be compared among couples taking into account covariates such as the average frequency of sexual intercourse. These predictions can be made for each couple in order to provide personal advices or specific drugs if there are predicted fertility problems. An additional aspect is that it can be feasible to investigate the effect of treatment on couples treated due to fertility problems by considering an extended version of the latent Markov model as recently proposed by Bartolucci et al. (41).

The analysis can be extended to consider the sex of the unborn child. The latent Markov model can also be extended considering the drop-out so as to compare the estimates obtained with the current approach. Our proposal may also be used to implement programs and devices by which each woman providing her daily CMS levels and her daily BBT may receive automatic signals related to her fertility or/and signals related to adverse health conditions.

## Ethics Statement

The research protocol related to the data collection was reviewed and approved by the Institutional Review Boards of Fondazione Lanza (Padua, Italy) and Georgetown University (Washington, DC, USA).

## Author Contributions

FP proposed the model to analyze the data and wrote the entire article. SZ helped to define the scenario of the application and to write some parts of the following Sections: 2, 3.1 and 4. MB was involved in the data collection. He helped to define the rule of the C.A.Me.N. symphothermal method and to deal with outliers in the historical data.

## Conflict of Interest Statement

The authors declare that the research was conducted in the absence of any commercial or financial relationships that could be construed as a potential conflict of interest.

## Footnotes

^1^see also http://www.confederazionemetodinaturali.it/titolo-diverso/s83b71096

^2^http://www.creightonmodel.com

^3^For additional details on Prof. Bernardo Colombo see also https://it.wikipedia.org/wiki/Bernardo_Colombo
